# Phase II multicentre randomised study of docetaxel plus epirubicin *vs* 5-fluorouracil plus epirubicin and cyclophosphamide in metastatic breast cancer

**DOI:** 10.1038/sj.bjc.6602179

**Published:** 2004-09-21

**Authors:** J Bonneterre, V Dieras, M Tubiana-Hulin, P Bougnoux, M-E Bonneterre, T Delozier, F Mayer, S Culine, N Dohoulou, B Bendahmane

**Affiliations:** 1 Centre Oscar Lambret, 3 rue Frédéric Combemale, 59020 Lille, France; 2 Institut Curie, Paris, France; 3 Centre René-Huguenin, Saint-Cloud, France; 4 CHU Bretonneau, Tours, France; 5 Centre François Baclesse, Caen, France; 6 Centre Georges-François Leclerc, Dijon, France; 7 Centre Val d'Aurelle, Montpellier, France; 8 Polyclinique Bordeaux Nord, Bordeaux, France; 9 Laboratoire Aventis, Paris, France

**Keywords:** docetaxel, epirubicin, cyclophosphamide, 5-fluorouracil, metastatic breast cancer, first-line chemotherapy

## Abstract

The purpose of the study was to evaluate the efficacy and safety of docetaxel plus epirubicin (ET) and of 5-fluorouracil plus epirubicin and cyclophosphamide (FEC) as first-line chemotherapy for metastatic breast cancer. A total of 142 patients (intent-to-treat (ITT)) with at least one measurable lesion were randomised to receive docetaxel 75 mg m^−2^ plus epirubicin 75 mg m^−2^ or 5-fluorouracil 500 mg m^−2^ plus epirubicin 75 mg m^−2^ and cyclophosphamide 500 mg m^−2^ intravenously once every 3 weeks for up to eight cycles. Prophylactic granulocyte-colony-stimulating factor was only permitted after the first cycle, if required. Per-protocol analysis (*n*=132) gave an overall response rate for ET of 63.1% (95% confidence interval (CI), 50–78%) and for FEC 34.3% (95% CI, 23–47%) after a median seven and six cycles, respectively. Intent-to-treat population (*n*=142) gave an overall response rate for ET of 59% (95% CI, 47–70%) and for FEC 32% (95% CI, 21–43%) after a median seven and six cycles, respectively. The median response duration for ET was 8.6 months (95% CI, 7.2–9.6 months) and for FEC 7.8 months (95% CI, 6.5–10.4 months). The median time to progression (ITT) for ET was 7.8 months (95% CI, 5.8–9.6 months) and for FEC 5.9 months (95% CI, 4.6–7.8 months). After a median follow-up of 23.8 months, median survival (ITT) for ET and FEC were 34 and 28 months, respectively. Nonhaematologic grade 3–4 toxicities were infrequent in both arms. Haematologic toxicity was more common with ET and febrile neutropenia was reported in 13 patients (18.6%) in the ET group. Two deaths in the ET group were possibly related to study treatment. In conclusion, both ET and FEC were associated with acceptable toxicity. ET is a highly active first-line therapy for metastatic breast cancer.

Only a small number of patients with metastatic breast cancer achieve long-term survival (complete remission for more than 5 years in 3.1% of patients in one survey) (Greenberg *et al*, 1996). The median survival of women with hormone-insensitive advanced breast cancer remains at around 2 years, and the chief aims of management continue to be prolonging survival and easing of symptoms, minimising treatment toxicity, and optimising of quality of life (Perez, 1999).

Treatment of metastatic breast cancer usually involves hormone therapy and/or chemotherapy with or without a biologic agent (eg trastuzumab in patients with tumours overexpressing HER2). Radiation therapy and/or surgery may be indicated for patients with limited, but symptomatic metastases. Cytotoxic chemotherapy is usually recommended for patients whose tumours have progressed on hormonal therapy, those with hormone receptor-negative tumours, and those with visceral metastases (National Cancer Institute, 2002).

The experience of two decades of clinical studies has shown that, in general, the use of combination chemotherapy is associated with higher response rates than single-agent treatment in metastatic breast cancer (Fossati *et al*, 1998). As recently reviewed (Crown *et al*, 2002; Nabholtz *et al*, 2002), the anthracyclines are well established in first-line chemotherapy for metastatic breast cancer, and anthracycline-containing regimens have been shown to confer higher response rates and longer survival overall than nonanthracycline combinations such as cyclophosphamide plus methotrexate and 5-FU (CMF) (A'Hern *et al*, 1993). First-line combinations include doxorubicin plus cyclophosphamide (AC), epirubicin plus cyclophosphamide (EC), 5-fluorouracil (5-FU) plus doxorubicin and cyclophosphamide (FAC), or 5-FU plus epirubicin and cyclophosphamide (FEC).

Of the novel chemotherapeutic agents introduced during the 1990s, the taxanes have emerged as some of the most active drugs in breast cancer. The semisynthetic taxoid docetaxel (Taxotere®) has been shown in phase III studies to be effective in metastatic disease previously treated with an anthracycline or alkylating agent (Chan *et al*, 1999; Nabholtz *et al*, 1999; Sjöström *et al*, 1999; Bonneterre *et al*, 2002). Docetaxel has shown substantial activity when combined with an anthracycline, with two notable phase III trials showing significantly higher tumour response rates with docetaxel plus doxorubicin than with AC, and with docetaxel plus doxorubicin and cyclophosphamide (TAC) than with FAC (Nabholtz *et al*, 2001, 2003).

Epirubicin is an anthracycline that may be less cardiotoxic than doxorubicin, and noncomparative phase II studies have shown very encouraging responses to treatment when this drug is combined with docetaxel as first-line therapy in metastatic breast cancer (French Epirubicin Study Group, 1998; Airoldi *et al*, 2001; Morales *et al*, 2002; Pharmacia Corporation, 2002; Yeo *et al*, 2002). The present randomised phase II study evaluated epirubicin 75 mg m^−2^ plus docetaxel 75 mg m^−2^ (ET) and the standard FEC triple combination, using the same dose of epirubicin (75 mg m^−2^).

## MATERIALS AND METHODS

This prospective, randomised, open label study was carried out across 12 centres in France. The study protocol was approved by the ethics committee at each participating centre.

### Patients

Women aged 18 to 65 years were required to have histologically or cytologically proven breast cancer with metastases and at least one bidimensionally measurable lesion. Written informed consent was required from each patient before enrolment. Prior adjuvant or neoadjuvant therapy, with or without anthracyclines (maximum cumulative dose of ⩽310 mg m^−2^ for doxorubicin, 460 mg m^−2^ for epirubicin and 75 mg m^−2^ for mitoxanthrone), was allowed if completed at least 12 months before the study, as were adjuvant locoregional radiotherapy or hormone therapy if completed at least 4 weeks or immediately before study entry, respectively. Other requirements were a World Health Organization (WHO) performance status of ⩽2, left ventricular ejection fraction (LVEF) within the normal range for each institution, no symptomatic central nervous system metastases, and adequate haematologic, renal and liver function. Prior treatment with a taxane and previous chemotherapy for metastatic breast cancer was not allowed.

Patients were not eligible if they had localised or inoperable locally advanced disease, history of any other cancer (with the exception of nonmelanoma skin cancer or cervical carcinoma *in situ*), other significant medical conditions (most notably cardiac, neurologic, or psychiatric disorders), any uncontrolled infection, or sensory or motor neuropathy of severity greater than WHO grade 1. Patients receiving a corticosteroid at a dosage equivalent to 20 mg day^−1^ or more of methylprednisolone, those in whom corticosteroid treatment was contraindicated, and pregnant or breast-feeding women were excluded. Effective contraception was required for women of child-bearing age.

### Treatment plan

Patients were assigned according to a centralised predefined schedule with randomisation stratified according to centre to one of two treatment arms: epirubicin (Ellence®, Farmorubicin®) 75 mg m^−2^ over 10 min plus docetaxel (Taxotere®; Aventis, Antony, France) 75 mg m^−2^ over 1 h (ET), or 5-FU 500 mg m^−2^ over 1 h plus epirubicin 75 mg m^−2^ over 10 min plus cyclophosphamide 500 mg m^−2^ over 30 min (FEC). All agents were given by intravenous infusion once every 21 days for up to eight cycles, with a maximum allowable cumulative dose of anthracycline equivalent to doxorubicin 550 mg m^−2^. Prophylactic premedication with corticosteroids (six doses of methylprednisolone 40 mg or equivalent) was given for 3 days to all patients on the ET arm. Prophylactic antiemetic treatment was mandatory for all patients, with the addition of loperamide for moderate to severe diarrhoea if necessary.

### Assessment of response and toxicity

The pretreatment evaluation included a full medical history and physical examination, haematology and biochemistry, and visceral tumour assessment. The radiologic examination included front and lateral chest X-rays, abdominal ultrasound and/or computed tomography (CT) scan, systematic bone scan, and standard X-rays, CT scans, or magnetic resonance imaging of suspicious lesions. An electrocardiogram and echocardiography were performed in all patients at baseline and particularly in case of history of cardiac disease or prior cumulative dose of anthracycline or anthracenedione above the specified limits.

Physical examinations and liver function tests were repeated at the beginning of each treatment cycle. Assessments of left ventricular function with multiple gated acquisition scanning or echocardiography were performed every four cycles until the maximum cumulative dose of anthracycline was reached, and every two cycles thereafter. Haematologic analysis was carried out immediately before each treatment and on day 7 (with an additional test on day 14 in case of fever). Prophylactic granulocyte-colony-stimulating factor (G-CSF) was given if the absolute neutrophil count (ANC) decreased to <0.5 × 10^9^ l^−1^ for more than 7 days and/or the platelet count was reduced to ⩽25 × 10^9^ l^−1^ and/or febrile neutropenia was apparent (grade 4 neutropenia with concomitant temperature ⩾38.5°C, which required intravenous antibiotics or hospitalisation). If a second neutropenic episode occurred despite G-CSF, the dose of docetaxel in the ET group was to be reduced to 60 mg m^−2^ for all remaining cycles, with no further reductions allowed. In the event of a second episode in the FEC group, the dose of epirubicin was reduced to 60 mg m^−2^ until recovery to ANC ⩾1.5 × 10^9^ l^−1^. Treatment withdrawal was required for a third episode. Dose delay to a maximum of 2 weeks was allowed for patients with ANC <1.5 × 10^9^ l^−1^ and/or platelet count <100 × 10^9^ l^−1^ at day 22 (second cycle).

A 2-week dose delay was allowed for patients with grade 3 hyperbilirubinaemia, but treatment withdrawal was required for grade 4 hyperbilirubinaemia. Dose delay was also allowed for patients with abnormal hepatic enzyme levels. Patients were withdrawn from the study if anaphylaxis or a recurrent severe (National Cancer Institute Common Toxicity Criteria (NCI-CTC *version 2.0*) grade 4 hypersensitivity reaction was observed. Oral treatment with spironolactone 50 mg day^−1^ was permitted for fluid retention, with the addition of furosemide if necessary. Cessation of chemotherapy was required in patients showing signs of congestive heart failure, functional criteria for cardiotoxicity, or decrease of more than 10% from baseline in LVEF to below the lower limit of normal for the institution.

Target lesions were assessed every two cycles. All objective responses were confirmed at least 4 weeks after the initial response determination and reviewed by an independent committee of radiologists. A complete tumour assessment was carried out 28 days after the last infusion, with monthly follow-up thereafter for 6 months, then every 2 months for 1 year. Tumour responses in measurable and/or assessable lesions were assessed according to WHO criteria (World Health Organization, 1979). The best overall response was taken as the best response obtained from the start of treatment until disease progression. The duration of response was the time from complete (CR) or partial (PR) response to the time that recurrent or progressive disease or death was first noted. Survival was defined as the time from beginning treatment to the time of death from any cause or the date of last contact if death was not recorded before the cutoff date.

Adverse events were monitored from the start of chemotherapy to 30 days after the last infusion and were graded using the NCI-CTC criteria. Serious adverse events were those that resulted in death or were life threatening, required or prolonged hospitalisation, caused persistent or significant disability or incapacity, or were considered important medical events.

### Statistical methods

The primary objective of the study was to evaluate the efficacy of EC and FEC in terms of objective tumour response rate (ORR). Secondary end points included time to progression (TTP), duration of response, survival, and tolerability. Response rates were analysed for the intent-to-treat (ITT) population, which was defined as patients who had received at least one cycle of treatment and for the per-protocol population, which was defined as patients assessable for response who had received at least two cycles of chemotherapy. Time to progression and overall survival were calculated for the ITT population, which consisted of all patients who had received at least one cycle of study treatment.

Statistical analyses were carried out using SAS software Version 6.08. The TTP, response duration, and survival data were subjected to Kaplan–Meier analysis with censoring. The sample size as planned by protocol was based on an estimated ORR of 65% at the end of the eighth cycle. The number of assessable patients required per group to confer 95% power was calculated to be 88, and it was therefore planned to enroll 100 patients per arm.

## RESULTS

### Patients

A total of 142 patients were enrolled and randomised between September 1998 and November 2000, 70 to ET and 72 to FEC. The planned population was not included due to difficulties about enrolment. The respective assessable populations were 65 and 67. In the ET arm, one patient withdrew consent, there were three early deaths (one each due to sepsis, gastrointestinal haemorrhage, and a cause unrelated to treatment), and one patient had no assessable lesion. In the FEC arm, one patient withdrew consent and four patients had no assessable lesions. Patient and tumour characteristics were generally similar at baseline between groups, although the proportion of patients with an original diagnosis of stage IV disease was higher in the ET than in the FEC arm (33 *vs* 21%). In addition, more patients in the ET group showed metastatic involvement of the lung, liver, skin, bone, or more than three organs overall. The median age was 54 years, and 97% of patients had a WHO performance status of 0 to 1. Overall 49% of patients received previous adjuvant or neoadjuvant chemotherapy, 42% with an anthracycline-based regimen. A slightly higher proportion of patients in the FEC group had oestrogen or progesterone receptor-positive tumours (Table 1

**Table 1:** Patient and disease characteristics at baseline

	**No. of patients (%)**	
	**ET (*n*=70)**	**FEC (*n*=72)**
*Age (years)*		
Median	54	54
Range	34–73	23–71
		
*WHO performance status*		
0	43 (61)	52 (72)
1	25 (36)	17 (24)
2	2 (3)	3 (4)
		
More than three organs involved	45 (64)	32 (44)
		
*Type of lesion*		
Lymph node superficial	24 (34)	17 (24)
Lymph node deep	20 (29)	24 (33)
Breast	25 (36)	19 (26)
Lung	36 (54)	27 (38)
Liver	45 (64)	41 (57)
Skin	13 (19)	6 (8)
Bone	41 (59)	35 (49)
Other	9 (13)	2 (3)
		
*Stage at initial diagnosis*		
I	7 (10)	14 (19)
II	30 (43)	32 (44)
III	6 (9)	8 (11)
IV	23 (33)	15 (21)
Unknown	4 (6)	3 (4)
		
*SBR tumour grade*^a^		
1	5 (7)	6 (8)
2	42 (60)	32 (44)
3	17 (24)	24 (33)
Unknown	6 (9)	10 (14)
		
*Hormone receptor status*		
ER positive	38 (54)	48 (67)
ER negative	26 (37)	20 (28)
PR positifs	36 (51)	43 (60)
PR negative	28 (40)	25 (35)
Unknown	6 (9)	4 (6)
		
*Prior chemotherapy*		
None	37 (53)	35 (49)
Anthracycline based	29 (41)	31 (43)
Nonanthracycline based	4 (6)	6 (8)

ER=estrogen receptor; ET=epirubicin plus docetaxel; FEC=5-fluorouracil plus epirubicin plus cyclophosphamide; PR=progesterone receptor; SBR=Scarff–Bloom–Richardson; WHO=World Health Organization.

a Scarff–Bloom–Richardson system: grade tumour on the basis of glandular formation, mitotic rate, and nuclear pleiomorphism. Grade 1=well differentiated; grade 2=moderately differentiated; grade 3=poorly differentiated.

).

### Treatment administration

A total of 441 and 430 cycles of treatment were administered in the ET and FEC arms, respectively, with median cycle numbers per patient of 7 for ET and 6 for FEC (range, 1–9 for both ET and FEC). The median overall relative dose intensity was 1, and treatment was delayed by more than 7 days in 3% of cycles in both groups. Epirubicin dose reduction was required in 4.5% of ET and 0.7% of FEC cycles. Treatment was discontinued due to completion of therapy in 75 patients (42 ET and 33 FEC) and due to disease progression in 40 patients (14 ET and 26 FEC).

Poststudy treatments for ET and FEC arms, respectively, were as follows: surgery in 4.2 and 5.5% of patients, radiotherapy in 21.4 and 22.2%, hormonal therapy in 58.6 and 61.1%, and chemotherapy in 67.1% and 79.1. Second-line (during poststudy period) docetaxel was administered to 56.9% of FEC-treated patients and 8.6% of ET-treated patients.

### Response and survival

According to the per-protocol analysis, 65 patients in the ET group and 67 in the FEC group were evaluable for response (Table 2

**Table 2:** Clinical responses to chemotherapy (per-protocol analysis)

	**No. of patients (%)**	
	**ET (*n*=65)**	**FEC (*n*=67)**
CR	2 (3.1)	1 (1.5)
PR	39 (60.0)	22 (32.8)
Stable disease	20 (30.8)	36 (53.7)
Progressive disease	4 (6.2)	8 (11.9)
ORR^a^	41 (63.1, 95% CI, 50–78)	23 (34.3, 95% CI, 23–47)

**Figure 1:**
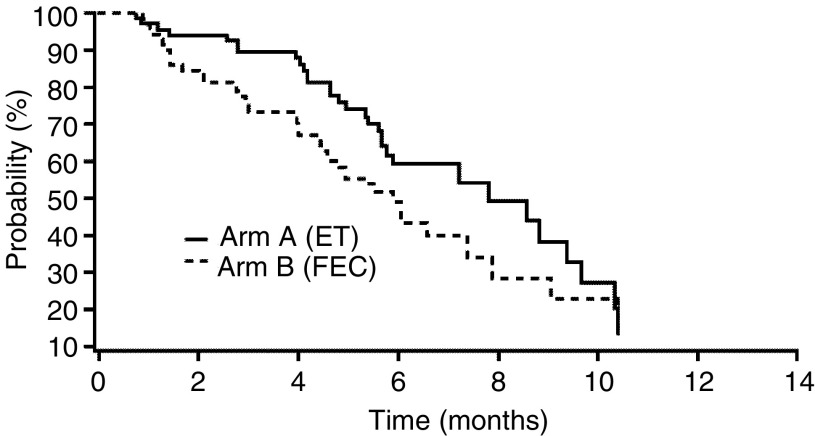
Time to tumour progression (ITT analysis).

CR=complete response; PR=partial response; ORR=objective response rate; CI=confidence interval; ET=epirubicin plus docetaxel; FEC=5-fluorouracil plus epirubicin plus cyclophosphamide.

a ORR=CR+PR.

). The ORR in each group was 63.1% (95% confidence interval (CI), 50–78%) with ET and 34.3% (95% CI, 23–47%) with FEC. According to the ITT population, the ORR was 59% (95% CI, 47–70%) with ET and 32% (95% CI, 21–43%) with FEC. CRs were reported in two patients receiving ET and one patient on FEC (Table 2). The median duration of response was 8.6 months (95% CI, 7.2–9.6 months) for ET and 7.8 months (95% CI, 6.5–10.4 months) for FEC at the cutoff date. The median TTP for the ITT population was 7.8 months (95% CI, 5.8–9.6 months) in the ET group and 5.9 months (95% CI, 4.6–7.8 months) in the FEC group (Figure 1

).

**Figure 2:**
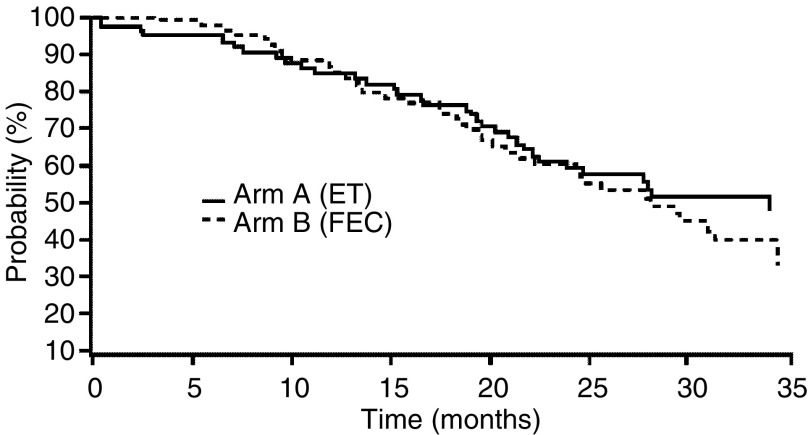
Overall survival (ITT analysis).

After a median follow-up of 23.8 months, median survival in the ITT population was 34 months in the ET group and 28 months in the FEC group; 37 (53%) and 31 (43%) patients, respectively, were still alive at the cutoff date (Figure 2

).

### Toxicity

The main NCI-CTC grade 3 to 4 toxicities were haematologic and are summarised in Table 3

**Table 3:** Grade 3 and 4 haematologic toxicity

	**No. of patients (%)**		**No. of cycles (%)**	
	**ET (*n*=70)**	**FEC (*n*=72)**	**ET (*n*=441)**	**FEC (*n*=430)**
Neutropenia	47 (67.1)	43 (59.7)	133 (30.2)	95 (22.1)
Febrile neutropenia	13 (18.6)	0	21 (4.8)	0
Leukopenia	31 (44.3)	24 (33.3)	101 (22.9)	47 (10.9)
Anaemia	3 (4.3)	1 (1.4)	3 (0.7)	1 (0.2)
Thrombocytopenia	2 (2.9)	1 (1.4)	2 (0.5)	1 (0.2)

ET=epirubicin plus docetaxel; FEC=5-fluorouracil plus epirubicin plus cyclophosphamide.

. Febrile neutropenia occurred in 13 patients (18.6%) in the ET group, and of the four patients who received G-CSF after the first neutropenic episode, only one experienced a second episode. Two patients in the FEC group received prophylactic G-CSF. Per-patient incidences of neutropenia were similar between groups, although this adverse event was seen during more cycles of ET than FEC (Table 3).

Nonhaematologic adverse events possibly or probably linked to study treatment were infrequent in both groups (Table 4

**Table 4:** Grade 3 and 4 nonhaematologic toxicity

	**No. of patients (%)**		**No. of cycles (%)**	
	**ET (*n*=70)**	**FEC (*n*=72)**	**ET (*n*=441)**	**FEC (*n*=430)**
Alopecia	38 (54.3)	24 (33.3)	47 (10.7)	30 (7.0)
Asthenia	7 (10.0)	4 (5.6)	10 (2.3)	4 (0.9)
Vomiting	6 (8.6)	6 (8.3)	9 (2.0)	10 (2.3)
Nausea	5 (7.1)	6 (8.3)	7 (1.6)	13 (3.0)
Infection	3 (4.3)	0	3 (0.7)	0
Diarrhoea	2 (2.9)	0	2 (0.5)	0
Sensory neuropathy	2 (2.9)	0	2 (0.5)	0
Fever	1 (1.4)	0	1 (0.2)	0
Nail toxicity	1 (1.4)	0	1 (0.2)	0
Skin toxicity	1 (1.4)	0	1 (0.2)	0
Cardiotoxicity	1 (1.4)	0	1 (0.2)	0
Haemorrhage	1 (1.4)	0	1 (0.2)	0
Abdominal pain	1 (1.4)	0	1 (0.2)	0
Pneumopathy	1 (1.4)	0	1 (0.2)	0
Stomatitis	0	3 (4.2)	0	3 (0.7)

ET=epirubicin plus docetaxel; FEC=5-fluorouracil plus epirubicin plus cyclophosphamide.

). Grade 3–4 alopecia, asthenia, nausea, and vomiting affected more than 5% of patients in either group. Serious adverse events considered by the investigators to be possibly or probably related to study treatment were more frequent in the ET group than with FEC (33 *vs* 3%). The majority of these events were accounted for by episodes of febrile neutropenia; others included asthenia, peripheral neuropathy, jugular vein thrombosis, skin infection, and decreased LVEF. Seven patients withdrew due to toxicity (five in the ET and two in the FEC group). As reported above, there were three early deaths in the ET group. Two of these deaths were potentially related to study treatment (one attributed to sepsis and one attributed to gastrointestinal haemorrhage occurring within 9 and 11 days after the last infusion, respectively). The third death was not related to study treatment, was of unexplained cause, and occurred within 9 days after the last infusion.

Overall, the proportion of patients who experienced reductions of at least 10 or 20% in LVEF was consistently higher with FEC than with ET, but grade 3–4 cardiac disorders occurred in two patients in the ET arm and not in the FEC arm.

## DISCUSSION

The present study shows that ET (epirubicin 75 mg m^−2^ plus docetaxel 75 mg m^−2^ every 3 weeks) is a highly active first-line treatment for metastatic breast cancer. Although comparison of the two arms was not prospectively planned, the results favour ET, particularly in terms of the ORR and median TTP relative to the established anthracycline-based triple combination of FEC (with the same epirubicin dose of 75 mg m^−2^). The ORR in the ET group was almost twice that of FEC (63.1 *vs* 34.3%), with two CR after ET treatment (compared with one CR with FEC). It should be noted that the tumour response rate with FEC in this study was somewhat lower than that reported in some other trials (Del Mastro *et al*, 2001; Baldini *et al*, 2003), but the population included in these trials presented a locally advanced breast cancer, although these response rates (41%) were consistent with those reported in a recent phase III study (Capotorto *et al*, 2003) of the same FEC regimen.

The median TTP was 7.8 months (95% CI, 5.8–9.6 months) for ET and 5.9 months (95% CI, 4.6–7.8 months) for FEC. The median survival was 34 months for ET and 28 months for FEC, although the survival curves appeared similar. However, this outcome may have been affected by the unplanned use of second-line (during the poststudy period); 56.9% of FEC-treated patients received subsequently docetaxel, whereas only 8.6% of ET-treated patients.

A prospectively designed and appropriately powered comparative trial, lacking potentially confounding late interventions, would be needed to investigate potential differences in survival.

Our results are consistent with other studies in which docetaxel has been combined with an anthracycline. Overall response rates of 57–79% were reported with 3-weekly combinations of docetaxel 60–80 mg m^−2^ in five first-line noncomparative studies in a total of 170 patients with metastatic breast cancer (Sparano *et al*, 2000; Baltali *et al*, 2001; Lippe *et al*, 2002; Aihara *et al*, 2003; Morales *et al*, 2004). Previous noncomparative studies of 3-weekly epirubicin 75 mg m^−2^ plus docetaxel 75 mg m^−2^ have yielded overall tumour response rates of 67–84%, with overall survival of up to approximately 2 years (Airoldi *et al*, 2001; Yeo *et al*, 2002; Morales *et al*, 2004).

Adverse events in the present study were predictable. Reversible myelosuppression was the dose-limiting toxicity in both treatment arms, although this was more common with ET than with FEC. Nevertheless, fewer than 5% of cycles in the ET group required dose adjustment (of epirubicin) or G-CSF support. Toxicity was higher overall with ET than with FEC, but frequencies and severities of adverse events were acceptable in the context of the disease being treated and the high response rate with ET, and were manageable in both groups. Alopecia and asthenia were the most common nonhaematologic toxicities. Neurosensory symptoms and cardiotoxicity were very infrequent; the incidence of sensory neuropathy reported (2.9%) can be compared to the incidence of around 4–5% with docetaxel 100 mg m^−2^ as monotherapy according to collated data from patients with metastatic breast cancer (Aventis Pharmaceuticals Inc., 2003).

Previous phase II studies have shown similar profiles and frequencies of adverse events when docetaxel is combined with an anthracycline. Haematologic adverse events (most notably neutropenia) were most common, with no excessive cardiac toxicity or any indication that the addition of docetaxel to doxorubicin increases the cardiotoxicity of the latter (Sparano *et al*, 2000; Baltali *et al*, 2001; Lippe *et al*, 2002; Aihara *et al*, 2003; Morales *et al*, 2004). In one phase II noncomparative trial of epirubicin and docetaxel, no grade 4 nonhaematologic adverse events and very low levels of cardiotoxicity (reversible congestive heart failure in one of 60 patients only) were observed (Yeo *et al*, 2002), whereas in another study there was no grade 3–4 cardiac toxicity and no treatment-related mortality among 46 patients (Airoldi *et al*, 2001). A further recent trial of 133 patients reported that two withdrew treatment due to cardiotoxicity, although none developed congestive heart failure (Morales *et al*, 2002).

The results of this study are also consistent with final data from a recent phase III comparison of docetaxel 75 mg m^−2^ plus doxorubicin 50 mg m^−2^ (AT) *vs* doxorubicin 60 mg m^−2^ plus cyclophosphamide 600 mg m^−2^ (AC) in 429 patients with metastatic breast cancer in which the ORR was significantly higher with the former regimen (59 *vs* 47%; *P*=0.009) and the median TTP was superior for AT (37.3 *vs* 31.9 weeks with AC; *P*=0.014). The median survival in this study was similar at approximately 22 months in both arms (Nabholtz *et al*, 2003). Grade 3 and 4 neutropenia was frequent in both groups, although febrile neutropenia (33 *vs* 10%; *P*<0.001) and infection (8 *vs* 2%; *P*=0.01) were more common in patients receiving AT. These findings are consistent with those in the present study.

Of particular interest are the results of the large phase III first-line comparison of epirubicin 75 mg m^−2^ plus paclitaxel 200 mg m^−2^ *vs* epirubicin 75 mg m^−2^ plus cyclophosphamide 600 mg m^−2^ carried out by the UKCCCR in 705 patients (Carmichael, 2001). The ORR was 67% in the epirubicin plus paclitaxel arm and 57% with epirubicin plus cyclophosphamide. Interestingly, there was no significant difference between treatments in the median progression-free (6.5 *vs* 6.7 months; primary end point) and overall (13.7 *vs* 13.8 months) survival after a median follow-up of 15 months. Of the 705 patients, 70% received all six planned cycles, with equivalent epirubicin dose intensity in both arms. Dose reductions and delays were attributable chiefly to myelosuppression and infection, and 46 and 37%, respectively, of patients receiving epirubicin plus paclitaxel and epirubicin plus cyclophosphamide experienced grade 3–4 toxicity (excluding alopecia). Severe mucositis (6 *vs* 2%; *P*=0.02) and neurotoxicity (5 *vs* 1%; *P*=0.003) were more common in patients receiving epirubicin plus paclitaxel than in those receiving epirubicin plus cyclophosphamide. These findings may be compared with those of the present study, which showed grade 3–4 sensory neuropathy in 2.9% of patients receiving ET, with no grade 3–4 stomatitis. In the UKCCCR study, severe infection was observed in 14% patients receiving epirubicin plus paclitaxel and 11% of patients on epirubicin plus cyclophosphamide, which may be compared with an incidence of grade 3–4 infection of only 4.3% in ET patients in the present trial. Overall, therefore, contrast of the UKCCCR data with the results of the present study suggests a potential advantage of docetaxel over paclitaxel when either is combined with epirubicin for metastatic breast cancer, although comparative trials will be required to confirm this (Jones *et al*, 2003).

In summary, the combination of docetaxel plus epirubicin is a highly active first-line treatment for metastatic breast cancer with acceptable toxicity, particularly for patients with symptomatic nonindolent disease. Evaluation of this combination is already underway in the adjuvant setting.
